# Intelligent Manufacturing Technology in the Steel Industry of China: A Review

**DOI:** 10.3390/s22218194

**Published:** 2022-10-26

**Authors:** Dongdong Zhou, Ke Xu, Zhimin Lv, Jianhong Yang, Min Li, Fei He, Gang Xu

**Affiliations:** 1 Collaborative Innovation Center of Steel Technology, University of Science and Technology Beijing, 30 Xueyuan Road, Haidian District, Beijing 100083, China; 2 Yangjiang Alloy Material Laboratory, 1 Luoqin Road, Jiangcheng District, Yangjiang 529500, China; 3 School of Mechanical Engineering, University of Science and Technology Beijing, 30 Xueyuan Road, Haidian District, Beijing 100083, China

**Keywords:** intelligent manufacturing, steel industry, China, typical models

## Abstract

Intelligent manufacturing, defined as the integration of manufacturing with modern information technologies such as 5G, digitalization, networking, and intelligence, has grown in popularity as a means of boosting the productivity, intelligence, and flexibility of traditional manufacturing processes. The steel industry is a necessary support for modern life and economic development, and the Chinese steel industry’s capacity has expanded to roughly half of global production. However, the Chinese steel industry is now confronted with high labor costs, massive carbon emissions, a low level of intelligence, low production efficiency, and unstable quality control. Therefore, China’s steel industry has launched several large-scale intelligent manufacturing initiatives to improve production efficiency, product quality, manual labor intensity, and employee working conditions. Unfortunately, there is no comprehensive overview of intelligent manufacturing in China’s steel industry. We began this research by summarizing the construction goals and overall framework for intelligent manufacturing of the steel industry in China. Following that, we offered a brief review of intelligent manufacturing for China’s steel industry, as well as descriptions of two typical intelligent manufacturing models. Finally, some major technologies employed for intelligent production in China’s steel industry were introduced. This research not only helps to comprehend the development model, essential technologies, and construction techniques of intelligent manufacturing in China’s steel industry, but it also provides vital inspiration for the manufacturing industry’s digital and intelligence updates and quality improvement.

## 1. Introduction

The steel industry is one of the most commonly used green materials for its good accessibility, lower manufacturing cost, and widespread usage, which play an important role in our daily lives and industrial manufacture in modern civilization [1]. For instance, as a pillar of the national economy, it provides fundamental materials for contemporary building, transportation, bridges, vehicles, ships, household appliances, electric power, marine engineering, and other aspects in our daily life [2]. Simultaneously, the steel industry is dealing with considerable carbon dioxide emissions, poor working conditions, environmental pollution, safety difficulties generated by high temperatures and poisonous gases, high labor intensity for employees, and repetitive labor [3]. To address the aforementioned issues, large-scale steel firms have engaged in automation, information transformation, and upgrading during the last several decades, which has had a major driving influence on the steel industry’s production efficiency and automation level [4].

With the arrival of the new century and the progressive completion of industrialization and urbanization in China, the steel industry’s output has reached supersaturation [5]. Appearing to be intensely competitive, the industry is dealing with a scarcity of high-end items, a long new product R&D cycle, low worker efficiency, uneven quality, and overall low company profitability. This necessitates transforming the manufacturing model to provide clients with a more flexible bespoke product model while also shortening the product’s production cycle. How to promote the upgrading of product quality in the steel industry, improve intelligence, reduce the research and development cycle of new materials, and support the economy’s sustainable development are hot issues of concern to the entire society and can be addressed by utilizing the rapidly developing technologies of the Internet, artificial intelligence, and big data methods.

Intelligent manufacturing, defined as the integration of manufacturing with modern information technologies such as 5G, digitalization, networking, and intelligence, has grown in popularity as a means of increasing the productivity, intelligence, and flexibility of traditional manufacturing processes. It is currently one of the hotspots in worldwide research and industrial application, reflecting a significant shift away from the traditional production, manufacturing, and sales paradigm of the past and toward a customized production strategy oriented on customer needs. Governments around the world, led by developed countries such as Germany, United States, and Japan, have included intelligent manufacturing in their national development plans, with the goal of increasing the level of intelligence in the manufacturing industry, establishing an intelligent factory with personalized customization and optimal resource allocation that adapts to large specifications, and integrating customers and business partners in business and value processes to increase productivity.

On the basis of this concept, several experts have undertaken in-depth research on intelligent manufacturing. The infrastructure issues for the development of Industry 4.0 have all been introduced since the concept of Industry 4.0 was proposed for advancing manufacturing to realize short product life cycles and extreme mass customization in a cost-efficient manner [6]. These include the construction method [7], self-organization in the context of Industry 4.0 [8], standardization, integrated information system, training and education, and artificial intelligence applications [9]. Then, in Industry 4.0, the uses of intelligent manufacturing have been researched in a variety of industrial enterprises, including heavy industry [10], supply chain management [11], transforming production management [12], small- and medium-sized enterprises (SMEs) [13], and cyber-physical manufacturing metrology system (CP2MS) [14]. Ghobakhloo [15], Kamble [16], Osterrieder [17], Ching [18], Leng [19], and Chauhan [20] presented the 4.0 technologies utilized in sustainable industrial systems.

As a result, the digital twin is a new technology used in intelligent manufacturing that can understand the state of intelligent manufacturing systems in real time and predict system failures [21]. It has since emerged as the primary technology and tool for manufacturing industries to realize intelligent cyber-physical integration and digital transformation by leveraging these technologies [22]. The digital twin was described by Moeller et al. as a major enabler for the digital transformation of intelligent manufacturing [23]. By researching and evaluating the enhancement of the financial management model in the intelligent manufacturing model and the optimization of cost incurred, Tan evaluated the financial performance [24]. Wu analyzed the workings of a novel application framework for a digital-twin-driven ship intelligent manufacturing system [25]. Li et al. offered the digital-twin framework as the approach for the green performance evaluation of intelligent manufacturing [26]. It is a unique hybrid MCDM model based on fuzzy rough-sets AHP, multistage weight synthesis, and PROMETHEE II. Zhang suggested a collaborative framework based on digital-twin technology for complicated product design, production, and service integration [27]. Li conducted research on the need for conventional manufacturing companies to undergo a digital transformation before examining the digital innovation model for these companies using a case study of the “Internet Plant” of the Haier Group [28].

The framework, development, key technologies, and applications of BDA for intelligent manufacturing systems were discussed by Wang et al. [29]. In addition, the amount of data from manufacturing systems has been quickly growing due to the development of Internet of Things (IoT), 5G, and cloud-computing technologies. Deng conducted research on the spatial agglomeration and superior layout features of the intelligent manufacturing supply chain, which helps businesses and governments make decisions [30]. The supply chain, industry chain, cyber-physical system, big data, Internet of Things, cloud computing, industrial transformation, and value chain ideas were all considered by Ge et al. in their study of a typical intelligent manufacturing [31]. Li offered a theoretical analysis foundation for big-data-driven technology to direct decision-making in intelligent manufacturing, thoroughly establishing its viability in the field of intelligent manufacturing, including major benefits and internal motivation [32]. Guo et al. described an intelligent decision support system (DSS) based on data-mining technology that is applied to enterprises to create an Internet of Things based intelligent DSS for the manufacturing industry, supporting the decision-makers in making intelligent decisions through the intelligent DSS [33]. An intelligent workshop based on digital twinning was proposed by Yan et al. They first presented the theoretical model and system framework for the workshop’s digital twinning and then presented three important technologies for the workshop’s virtual simulation control [34].

Hu and colleagues examined the “perception analysis-choices” closed-loop mechanism of all data of the production process of electronic components using intelligent manufacturing technology based on industrial big data [35]. To make semiconductor production smarter, Ghahramani et al. performed a thorough investigation based on evolutionary computing and neural network techniques [36]. A paradigm for data-driven or intelligent manufacturing was created by Ma [37], Majeed [38], and Zhang [39] based on the demand response in various scenarios.

The primary areas of algorithm innovation in intelligent manufacturing’s big data analysis are process optimization, quality traceability, equipment problem detection, production enhancement and management efficiency, and lowering of labor intensity. Ben researched the key technologies for the radar complete machine’s intelligent assembly system, including virtual assembly for digital products, IoT-based intelligence in perception, intelligent material distribution, assembly line modeling and simulation optimization, and information system integration [40]. Wang et al. provided a summary of the diffusion model for implementing intelligent manufacturing in Hangzhou manufacturing enterprises. The impact of industrial policy accuracy on the spread of intelligent manufacturing applications is then examined using the improved SIR model and Matlab software [41]. The outstanding benefits of deep learning in intelligent-manufacturing-system modeling were detailed by Lan [42]. These benefits include an efficient method and strong tool for intelligent-manufacturing-system design, performance analysis, and running status monitoring; as well as a clear path for choosing, designing, or implementing the deep-learning architecture. Kong examined the effects of IoT technology on intelligent manufacturing and intelligent buildings, explained the integrated system framework for BIM technology (building information models), and suggested the system design for intelligent buildings and the architecture model for intelligent manufacturing based on IoT technology [43]. A cloud-assisted and edge-decision-making manufacturing architecture with production edges was introduced by Tang et al. [44]. Meanwhile, machine learning [45], the compound dual innovation capability model [46], the capability maturity model [47], the information model of a lithium ion battery intelligent manufacturing workshop [48], the neural network [49], the predictive maintenance method [50], and the multichain and data-chain partitioning algorithm [51,52,53] have also been studied to improve the intelligent methods in intelligent manufacturing.

More elements of intelligent manufacturing in addition to those that were mentioned above were also examined as being crucial to various intelligent manufacturing systems [54]. Time-saving under the revolutionary intelligent manufacturing cloud-control-systems architecture presented by Yan et al. was comprehensively analyzed [55]. Chen et al. [56] and Xing [57] examined the major technologies of intelligent manufacturing and robot application and forecasted their future development trend. In order to build intelligent process automation (IPA) in various sectors, Zhou et al. [58] and Lievano-Martinez [59] offered key ideas and suggested a framework for implementing IPA technology. Using the panel data of pertinent enterprises from 2011 to 2017 and the enterprise panel data of intelligent manufacturing implemented by China’s manufacturing industry from 2014 to 2019, Yang et al. [60] and Liu et al. [61] also looked into the impact of China’s intelligent policy on the performance of listed manufacturing companies. Furthermore, defect diagnosis and early warning for high-end equipment [62], deep integration of industrial artificial intelligence and the industrial Internet [63], the local workshop net [64], and the two-layer distributed hybrid industrial Internet of Things [65] have all been introduced.

Kang offered the intelligent manufacturing idea, the key system structure, and each key technology, and the future was predicted by conducting various evaluations on the application fields and technical development levels addressed [66]. Mittal and Ren [67,68] created an intelligent manufacturing framework based on big data analytics, including critical technologies and the notion of omnipresent servitization throughout the life cycle. Furthermore, a significant quantity of production process, equipment, quality, logistics, and other data is generated during the manufacturing process. Various types of industrial processes have traditionally had different operation objectives and modes. Guo suggested a self-adaptive collaborative control (SCC) mode for intelligent production-logistics systems to increase intelligence, flexibility, and resilience by utilizing cyber-physical systems (CPSs) and the industrial Internet of Things (IIoT) to address the aforementioned issues [69]. Tao and Xia [70,71] looked at the significance, application prospects, and research problems of digital-twin-driven intelligent manufacturing in the context of Industry 4.0.

Intelligent manufacturing, obviously, has a complete shape in both theory and reality, and it has steadily become a way of boosting production efficiency and product quality, cutting labor costs, and optimizing production processes. However, the steel industry has challenges such as a vast volume of data, a big number of equipment, a poor automation foundation, an incomplete mechanism model, and hysteresis in crucial process parameter detection. The steel industry’s production can be created to examine the relevant model in the aforementioned specific conditions. Yin proposed that the intelligentization of steel production lines should be based on the physical input and output of the geophysical system’s material, energy, and information flow networks to optimize the physical system structure of the manufacturing process and integrate the digital information system to achieve the intelligence state [72]. Yao [73], Liu [74], and Li [75] also suggested a steel intelligent factory composition analysis, steel process big data, steel production value chain restructuring, and artificial intelligence to boost intelligent manufacturing in China’s steel industry. The goal is to shape the system value chain between manufacturing processes and artificial intelligence. Yuan and Yin discussed the aims, characteristics, and routes of intelligent growth for the process manufacturing industry in 2035, as well as how to implement self-perception, self-learning, self-decision-making, self-execution, and self-adaptation in individual processes [76]. Iannino [77,78] studied a CPS-based simulation platform for long manufacturing facilities, as well as a hybrid peer-to-peer architecture for agent-based steel manufacturing processes. Govender [79], Shin [80], and Zheng [81] investigated the approach for implementing an Industry 4.0 framework and a human–cyber-physical system for production and operation decision optimization in the steel industry.

Over the previous three decades, China’s steel industry has made considerable progress in automation and information technology, which has considerably increased production efficiency, product quality, and company operation and management levels. With the rapid expansion of domestic steel firms in terms of production capacity, as well as the rapid upgrading of technology and equipment, China’s steel production now accounts for about half of the global total. However, new issues such as “large but not strong” and overcapacity, an insufficient supply of high-end items, long development cycles for new products, low labor efficiency, uncertain quality, and low company profitability continue to be a concern. As a result, China’s industry sees intelligent manufacturing as a key means of addressing the aforementioned issues. Unfortunately, no thorough complete introduction to the literature on intelligent manufacturing in China’s steel industry exists. We began this research by summarizing the construction goals and overall framework for intelligent manufacturing in China. Following that, we offered a brief review of intelligent manufacturing for China’s steel industry, as well as descriptions of two typical intelligent manufacturing systems. Finally, some major technologies employed for intelligent production in China’s steel industry were introduced. This research not only helps to comprehend the development model, essential technologies, and construction techniques of intelligent manufacturing in China’s steel industry, but it also provides vital inspiration for the manufacturing industry’s digital and intelligence updates and quality improvement.

## 2. Analysis of Intelligent Development of Steel Industry of China

### 2.1. General Briefing on Intelligent Manufacturing in the Steel Industry of China

**Figure 1:**
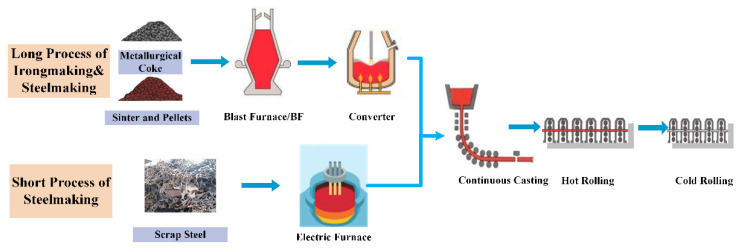
Schematic diagram of different processes in steel industry.

Figure 1 illustrates the two primary production models that are typically used in the steel industry: the long process of ironmaking and steelmaking procedure (LPISP) and the short process of steelmaking procedure (SPSP). The burden materials utilized in the LPISP are very different from those used in the SPSP. For instance, metallurgical coke, sinter, and pellets are used in the LPISP, while the SPSP uses scrap steel. In addition, the smelting furnace used in the steelmaking process differs depending on whether it is a converter or electric furnace (LPISP vs. SPSP).

Since 2015, China’s steel industry has begun to conduct small-scale intelligent transformation pilots to eliminate data islands, promote data sharing between processes, improve the quality control and traceability efficiency of the product production process, improve equipment fault diagnosis capabilities, improve energy efficiency, reduce logistics costs, reduce nonessential post staff, reduce the R&D cycle of new products, and reduce management. Table 1 shows the typical intelligent manufacturing projects used in China’s steel industry in recent years, which are mostly dispersed in the mine, rolling, and steelmaking and rolling processes, with the majority of them being carried out in the rolling procedure.

On the one hand, the physical changes in materials during the rolling process can be easily defined by control models and detection methods; and the level of automation, informatization, and modeling is higher than in the smelting operation. On the other hand, as the final step in the steel production process, the problems that occurred during the previous smelting and continuous casting procedures must be corrected and eliminated from the rolling link in a timely manner. Otherwise, the product quality and brand of the entire steel production line will suffer, causing significant harm to the enterprise. Decision-makers at steel businesses, logically, are also focused on intelligent manufacturing in the rolling process.

China developed the “Guidelines for the Construction of the National Intelligent Manufacturing Standard System”; researched the fundamental similarities, key technologies, key industry standards, and specifications of intelligent manufacturing; built a standard test verification platform; carried out technical specifications and standards throughout the entire process of test verification; and actively promoted all manufacturing-related fields. First, we discussed some fundamental rules and principles. Standard fundamentals include term definition, reference model, metadata, object ID registration, and parsing. Standards for management evaluation include the evaluation index system, measurement method, and implementation guide. Standards for information security included architecture, security, management, and evaluation. They also include adaptability to the environment, equipment dependability, and other criteria for quality. Second, we then discussed important technological norms and requirements., including equipment and product standards for industrial robots, industrial software, intelligent IoT devices, additive manufacturing, human–computer interface, and other technologies. We also discussed standards for network design, connectivity, and interoperability; convergence of fieldbus and industrial Ethernet; industrial sensor networks; industrial wireless systems; and industrial gateway communication protocols and interfaces. Standards for “smart factories” include network collaborative production, intelligent detection, intelligent logistics, and accurate supply-chain management. Standards for the industrial big data and cloud include cloud services, data analysis, and quality. Industrial processes use energy efficiency analysis software standards and service-oriented manufacturing standards, such as personalized customization and remote operation and maintenance services. Third, we discussed important industry rules and standards. Digital workshop integration applications in typical discrete industries and smart factory integration applications in process industries each represent one of ten important areas of industry standards and requirements.

### 2.2. Aims for Intelligent Manufacturing

The steel industry, as a typical process industry, has many production procedures, a large number of pieces of equipment, a large amount of energy consumption, a large number of operators, and a large number of complex reactions, including chemical reactions during the smelting procedure and physical changes during the rolling procedure. The interweaving of various influencing elements provides a huge barrier to intelligent manufacturing of the whole steel production process, further complicating the quality, energy, and product management procedures. Specifically, in the steel industry, there are quality data islands in various manufacturing processes, process adjustment is complex and difficult to achieve, the product development cycle is long, and product quality control cannot be accurate to the process. Furthermore, quality control and traceability are difficult to implement, and the smelting process is largely reliant on manual experience. Intelligent manufacturing, as a new generation of information and communication technology and advanced manufacturing technology, can quickly respond to internal and external changes in enterprises and establish a new model of personalized customization and resource optimization configuration centered on customer needs by improving the manufacturing industry’s intelligence level.

Therefore, intelligent manufacturing has resulted in a significant transformation in the steel industry’s organizational structure, namely a transition from a production organization to a service-oriented manufacturing organization. As a result, the steel industry’s goals for intelligent manufacturing in China may be stated as follows. Manufacturing enterprise operations will shift from responsive to customized, with optimization shifting from local to global. While management is focused on backward and in-the-moment pre-event transformation to achieve flexible production, equipment control has shifted from automation to intelligence. The following four factors are the primary goals for the completion of intelligent manufacturing in China’s steel industry.

First and foremost, boost productivity. On the one hand, breaking down data silos within a single process and between multiple processes involving ironmaking, steelmaking, continuous casting, and steel rolling, as well as leading the substance, energy, and information flow, could be efficiently utilized to reduce defective product rates and eliminate production efficiency losses caused by data islands and information gaps. Robots, on the other hand, are being used in large numbers to replace humans in repetitive, harsh environments and labor-intensive positions, gradually merging the main control position, improving personnel efficiency and intelligence level, and real-time online monitoring and fault prediction maintenance of key equipment to reduce equipment failure rate and unplanned downtime.

Second, boost product quality. The production process status monitoring system will cover many dimensions such as raw materials, equipment, process parameters, and laboratory data, effectively avoiding the influence of abnormal working circumstances on the quality of the steel manufacturing process. Then, by raising the intelligence level of the production line, the equipment control accuracy and equipment failure online monitoring and early warning system may effectively avoid the occurrence of recurring quality mishaps. Finally, a quality monitoring and control system was implemented across the ironmaking and steelmaking processes to eliminate worker interference on product quality and increase the precision and dependability of quality control. In addition, product quality optimization was implemented; and digitization, networking, and intelligence of product quality control methods were improved through the traceability of the whole steel product life cycle.

Third, cut production expenses. An energy medium optimization system and an automatic batching system are built to achieve the automatic entry of molten iron and scrap into the furnace, minimize material preparation response time, optimize smelting rhythm, and reduce energy consumption. Then, to shorten the smelting time and enhance smelting efficiency, information technologies such as automated detection of molten steel components, intelligent blowing control, and automatic component analysis were used. Finally, lowering manufacturing costs via the use of robots and intelligent maintenance decreases the rate of faulty goods and increases the rate of worker productivity.

Fourth, reduce the development cycle of new products. A virtual simulation system for the entire ironmaking and steelmaking process is constructed, and the number of experiments for new product development is reduced by replacing the traditional experimental trial-and-error method with a digital process simulation system, thereby shortening the R&D cycle and improving product development efficiency.

Above all, following the aforementioned goals for intelligent transformation, the typical steel industry’s product research and development cycle is typically reduced by about 30%, the rate of product defects is decreased by 10–20%, the energy used to produce a ton of steel is decreased by 10%, the production cost is decreased by 10%, and the production efficiency is increased by 10–25%. From the standpoint of financial investment revenue, intelligent manufacturing is one of the key avenues for the development of the steel industry in the future. The investment in intelligent manufacturing of steel production lines is expected to pay for itself in 3 to 5 years. 

### 2.3. Framework for Intelligent Manufacturing

**Figure 2:**
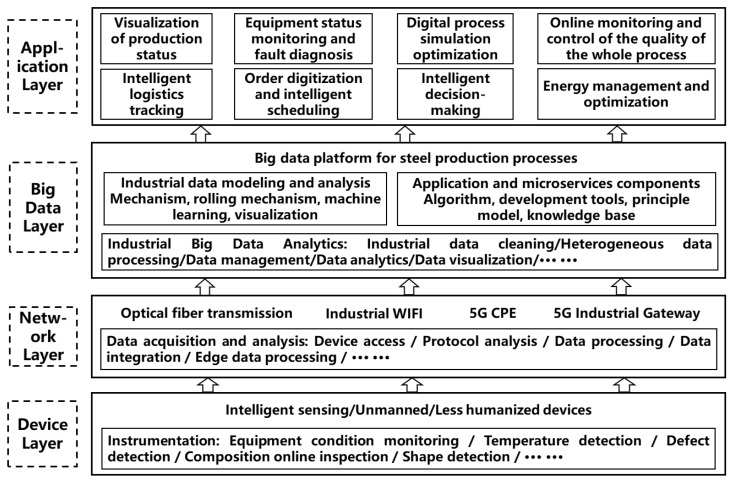
Framework for intelligent manufacturing in China’s steel industry.

As illustrated in Figure 2, the levels of the framework for intelligent manufacturing in China’s steel industry were split into the device layer (DL), network layer (NL), big data layer (BDL), and application layer (AL).

DL: The equipment parameters produced in the primary system, secondary system’s control model parameters, and tertiary system’s quality control parameters should be gathered in a timely and accurate manner during the overall ironmaking and steelmaking processes; particularly those affecting production efficiency, cost, and quality, such as key raw material parameters, process parameters, and quality process parameters. Equipment condition monitoring, temperature detection, defect detection, composition online inspection, shape detection, and other critical process parameters should all be detected online. Thus, the DL might provide a vast amount of raw data, laying a robust data basis for future big data applications and intelligent manufacturing.

NL: Due to the steel process’s big-scale, multi-equipment, and long process features, the physical distance between each piece of equipment is rather long, and huge data in diverse formats must be transmitted in real time across a significant number of networks. Not only this might help to enhance the manufacturing process’s reaction time, but it also could help to support BDL and AL applications. Therefore, data transport is mostly accomplished using optical fiber transmission, industrial Wi-Fi, 5G CPE, and a 5G industrial gateway. Data capture and analysis are dependent on device access, protocol analysis, data processing, data integration, and edge data processing.

BDL: Similar to the function of the data middle platform, the main objective of the BDL is to supply data sources and various application components for the application layer, as with the preceding data provision, data acquisition, and preprocessing. Thus, the BDL for steel production processes includes industrial data modeling and analysis, as well as application and microservices components. The former primarily consists of mechanism, rolling mechanism, machine learning, and visualization, whereas the latter provides the algorithm, development tools, primary model, and operating knowledge. Furthermore, the goals of big data analytics in the steel industry are mostly focused on industrial data cleansing, heterogeneous data processing, data management, data analytics, and data visualization.

AL: The primary goal of the AL is to realize real-time process diagnosis and optimization of the steel production process using a large number of data such as procedure, equipment, quality, logistics, order, and scheduling. In order to effectively improve production efficiency and the quality of the steel product, the rate of defective products and production costs should be reduced. Normally, a typical framework for intelligent manufacturing includes systems such as the visualization of production status system, equipment status monitoring and fault diagnosis system, digital process simulation–optimization system, online monitoring and control of quality in the entire ironmaking and steelmaking system, intelligent logistics tracking system, order digitization and intelligent scheduling system, intelligent decision-making system, energy management and optimization system, and intelligent decision-making system.

### 2.4. Sensors and Hardware for Intelligent Manufacturing

The sensors and hardware are the fundamental data resources and executors used in the steel industry’s process control and optimization on the basis of intelligent manufacturing. In general, Table 2’s classification of the sensors into general sensors, high-temperature application sensors, rolling testing sensors, and quality testing sensors of steel semi-finished products or finished products illustrates how they can be used in various processes.

Process design software, process simulation software, industrial control software, production management software, quality control software, equipment operation and maintenance software, energy management software, intelligent decision-making software, production and marketing integration software, and business control software are the main pieces of hardware used in the steel industry for intelligent manufacturing. Computer-aided (CAX) software; data-driven 3D design and modeling software; numerical analysis and visual simulation software; modular design tools; and specific expertise in the smelting process, models, nonstandard product processes, and standard databases make up the process design and simulation software. Additionally, the most frequently used software includes business intelligence software (BI), supply-chain management software (SCM), manufacturing execution system (MES), enterprise resource management (ERP), and product life-cycle management (PLM).

## 3. Typical Models for Intelligent Manufacturing of Steel Industry

### 3.1. Rolling Process Intelligent Manufacturing Model

The majority of China’s rolling steel production lines’ existing information islands are caused by an incomplete application of big data technologies, as well as a lack of a whole-process big data control system due to lacking inspection and testing technology, automation, and intelligent manufacturing equipment. As a result, the rolling process retains a low level of automation and intelligence, resulting in high labor costs and low labor efficiency, as well as inefficient quality control, testing, and traceability systems and a high proportion of faulty goods.

Based on the aforementioned issues, intelligent manufacturing for the rolling process primarily focuses on the following factors. To begin, the rolling mode employs important technologies such as industrial Internet data integration, hybrid model and data analysis, multi-objective interaction optimization, and intelligent robots. The unmanned slab storage, entire process quality monitoring, intelligent inspection, robot, and other intelligent applications were then used. This primarily concerned process control, material energy synergy optimization, and labor efficiency enhancement. It has significantly improved the intelligent control, predictive and early-warning forward-looking response, and multi-objective optimization level of business collaboration, as well as ultimately improved the manufacturing stability and flexibility of the hot-rolled production line and lowered manufacturing costs.

### 3.2. Steelmaking and Rolling Process Intelligent Manufacturing Model

In contrast to the intelligent manufacturing model for a single process in the rolling procedure at the workshop level, the steelmaking and rolling intelligent manufacturing model involves a number of procedural interfaces such as steel smelting and refining, continuous casting, and rolling procedure. Now, most of the issues that need resolving with China’s steelmaking and rolling systems are as follows. Due to the imperfect controlling model during the smelting process, the automation, informatization, and intelligence of the steelmaking system are insufficient. Furthermore, important operation parameters such as flue gas composition, molten steel temperature, billet temperature, molten steel and slag composition, and casting blank internal impurities are not accurately online-detected in time, causing the refining models to not achieve an implement control system via a closed-loop way.

The essential operational parameters as well as the quality of hot and cold rolled components cannot be detected online, which could not completely satisfy the unmanned or less humanized equipment and workshop due to a lack of intelligent packaging, intelligent grinding roller room, intelligent slab storage, and intelligent final product storage. In addition, intelligent robot operation has not yet been attained in operation positions under the conditions of high repeatability, high risk, and labor loss. Moreover, the product development cycle is lengthy and costly, with the majority of them relying on trial-and-error approaches, and there is a scarcity of simulation systems and tools that combine simulation. In conclusion, there are several date islands in the equipment management information system, and there is a data and function overlap across the systems.

The intelligent manufacturing project should be used in the steelmaking and rolling model to promote industrial structure adjustment, adhere to innovation-driven development, and finally realize the deep integration of informatization and industrialization to solve the problem of data islands between various procedures, improving the globalization level of quality control and production control, and finally realizing the deep integration of informatization and industrialization. Thus, the primary aim in steelmaking and rolling process intelligent manufacturing model is to establish horizontal and seamless integration of the whole industrial chain system of R&D, procurement, manufacturing, marketing, and service. The second goal is to achieve vertical and seamless integration between the information system and underlying automation system. The third goal is to achieve seamless end-to-end integration of workshop- and enterprise-level information systems. The fourth goal is to accomplish information exchange between upstream and downstream supply-chain firms. The steelmaking and rolling model in China was used to investigate the following concerns in particular.

- Intelligent sensing system. The monitoring of important process parameters such as steelmaking converters, refining furnaces, continuous casting ladles, and continuous casting machines is critical to optimizing the control model and increasing the degree of intelligence. Sensors, intelligent cameras, radio-frequency identification, and gateways are commonly used; and key technologies such as high-temperature heat pipes, image recognition, and voice recognition are integrated to create a comprehensive collection of production data that includes equipment data, product identification data, and factory environmental data to settle the demand for real-time awareness of the manufacturing process, operating data, and the status of important equipment. Simultaneously, in order to improve real-time sensor data transmission, it should be outfitted with high-performance network equipment with high system capacity, high transmission rate, multiple fault-tolerant mechanisms, and low latency, as well as using distributed industrial control networks, building software-defined agile networks, and realizing network optimized resource allocation.
- Centralized monitoring and controlling system. Integrate the control systems of major steelmaking and continuous casting processes, such as the converter area, refining area, continuous casting area, heating furnace area, and rolling area, and set up a production line monitoring system based on data collection. This system provides real-time monitoring of the manufacturing process, remote centralized control of equipment, and abnormal alert reminders, minimizing on-site operators and inspection staff, lowering labor intensity, and maintaining product safety.
- Production management and intelligent scheduling system. To realize real-time monitoring, balance coordination, and decision-making functions, it should build a production organization and intelligent scheduling system based on raw and fuel conditions, equipment status, and field-of-view requirements with plan execution, resource utilization, statistical analysis of output and quality, optimal scheduling of stable operating conditions, dynamic scheduling of abnormal operating conditions, as well as auxiliary production scheduling and decision-making functions.
- Intelligent device administration system. It should begin with equipment life-cycle state monitoring, tracking, and information maintenance throughout the whole equipment planning, design, production, procurement, installation, operation, maintenance, upgrading and transformation, and scrapping process. Then, create a full equipment status database by using big data analysis, artificial intelligence, virtual reality, and other technologies and use the main core equipment to produce a simulation model to achieve equipment failure early warning, alarm, and prediagnosis. Finally, create a standardized information collecting and control system, automatic diagnosis system, fault prediction model based on an expert system, and knowledge base of fault indexes. This should realize remote unmanned control, early warning of a hazardous working environment, monitoring of operational state, fault diagnostics, and self-repair.
- Quality controlling system. The quality management idea relates to information management, and a quality management system with quality standard maintenance, quality monitoring, inspection and laboratory testing, statistical analysis, and quality optimization should be built. Then, the product quality and operation parameters of the entire product manufacturing process are integrated by using big data analysis and machine-learning methods, which could accomplish the online judgment of product quality and the quality traceability analysis of the entire process. Finally, the important quality characteristics in the steelmaking and rolling processes are investigated, and the completed product quality could be obtained through online statistics, diagnosis, prediction, analysis, and optimization to improve the stability of product quality.
- Process simulation and prediction system. When combined with the actual status of the steelmaking procedure, it is difficult to coordinate this process because of the considerable production fluctuation and difficult precise control. First and foremost, the value of the production database is deeply excavated when combined with expert knowledge of the smelting process and on-site operation experiences. While the empirical model of the metallurgical process is established by using statistical analysis, machine learning, big data analysis, and other technical means, the model generalization ability is continuously improved through model training, the smelting production experience is mathematically expressed, and the decision optimization system related artificial intelligence ingredients are built to achieve the operation guidance and prediction of the actual production process. Then, comprehensive simulation calculations of fluid mechanics, chemical reaction, heat and mass transfer, and other simulation calculations on the equipment of the steelmaking converter procedure are performed by using the combination of a mechanism model and a data model. Similarly, the simulation model of the melting processes, continuous casting process, and rolling process should also be constructed. Finally, the actual steel procedure and virtual system interact in real time, which could optimize the manufacturing operation parameters during steelmaking and rolling procedures.
- An early warning system for employee safety. The entire process of tracking and managing employees visiting the manufacturing areas should be implemented by using satellite location, Wi-Fi, 5G, and other communication technologies, as well as intelligent wearable gadgets. Then a personnel management system should be created that can automatically perceive and obtain basic personnel information, personnel location, safety status, surrounding environment operation process information, statistical analysis of operation process data, and real-time grasp of personnel location trajectory and personnel position status. The system can automatically pop up alarm information and corresponding monitoring screens, as well as push the relevant reminder information to the relevant posts or personnel when entering the dangerous zone; or when key equipment abnormalities, major hazard source abnormalities, or other situations occur, achieving the aim of online monitoring, intelligent analysis, and linkage alarm to maintain employees’ safety.

## 4. Key Technologies for Intelligent Manufacturing in Steel Industry

### 4.1. Online Detection Technologies

Online detection in steel firms has an impact on accuracy and intelligence control [82]. The first issue is that crucial parameters of the manufacturing process cannot be identified in a timely manner, requiring smelting control to rely on professional knowledge and, on occasion, batch-quality incidents. The second issue is a lack of or obsolete terminal product quality detection systems, resulting in low efficiency and difficulties in successfully assuring the quality of intermediate or semi-finished steel products [83,84]. The third issue is that the basic data detection for intelligent transformation is either delayed or absent, resulting in a lack of vital parameters in the quality control system and the big data platform.

Detecting key parameters of the steel industry is vital in steel manufacturing to assure product quality and boost production efficiency. Currently available detection technologies can only identify the esthetic quality of steel products, such as dimensional correctness, plate shape, and surface defects. While the compositions of hot metal and slag during ironmaking and steelmaking procedures could only be detected offline, tissue performance and internal quality could not be detected for lacking a closed cycle of quality control of steel products. At the same time, there are some key parameters in the steel industry that cannot be detected online, such as raw material particle size and its compositions, high-temperature liquid slag temperature and its compositions, temperature [85], surface and internal defects of high-temperature plate strip, gas composition, automated detection of billet spray number characters, and product size. Furthermore, the bulk of detection tests are nonstandardized, and the steel industry, such as other domestic steel industries, must overcome certain critical production criteria and product quality characteristics in terms of testing technology.

**Figure 3:**
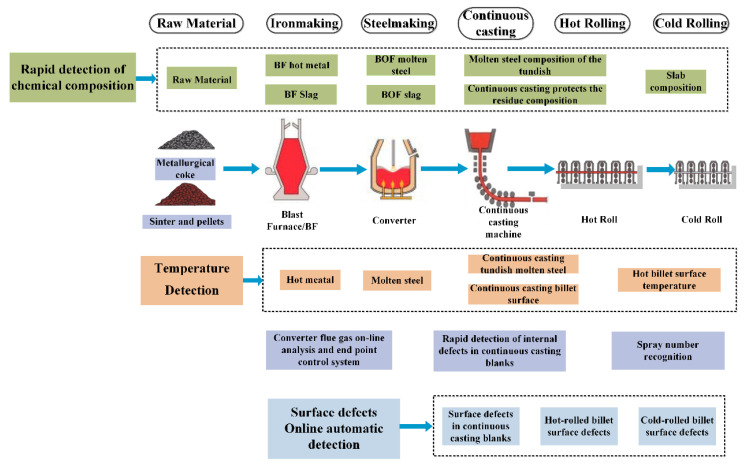
Online detection technologies used in China’s steel industry.

Figure 3 illustrates online detection technologies used in China’s steel industry. The detection technology development direction is to use advanced modern detection technologies such as machine vision [86,87,88,89,90,91,92,93], laser-induced breakdown spectroscopy (LIBS) [94,95,96,97,98], ultrasonic microscopy technology, and others, in conjunction with deep-learning algorithms and statistical modeling theory; to apply or develop intelligent perception technology on the production line; and to conduct online or rapid detection of key parameters throughout the manufacturing process. The ultimate purposes of online detecting technologies are to provide intelligent management and process optimization in the steel industry, improve the quality of terminal products, increase labor productivity and reduce labor costs, and provide vital fundamental data for quality control and big data platforms.

### 4.2. Quality Controlling Technology for the Entire Procedure of Steel Industry

The steel industry’s quality controlling system bridges the information gap between different procedures that involve ironmaking, steelmaking, rolling, and others; allows for the recording of information from the product’s entire manufacturing process; and provides platform support for quality improvement, quality design, research, development, and optimization. The enterprise’s existing data acquisition system is integrated, and high-precision real-time capture of production process data is achieved to meet data acquisition variables and acquisition frequency needs depending on business expectations. For instance, the following issues are the key benefits of quality control technology for the complete steel manufacturing operation [99].

To begin, the steel industry will be provided with an essential production process parameter and product quality monitoring [100], as well as online product quality grading, digital scoring values, and product quality grades. Then, by establishing a product quality prediction model, it will be possible to identify and be notified of potential quality concerns that are difficult to detect online, as well as conduct pre-control activities as soon as feasible. Furthermore, one should bring forward flow control suggestions for steel goods with potential quality problems or items in procedures as soon as possible to avoid unqualified products to be entered into the next production line, which ensures product quality stability in each production procedure through quality control [101]. This system collects data from any point in the manufacturing process, as well as diverse process data from associated processes, and automatically detects data. Finally, data analysis and process improvement should be started, and intelligent algorithms are used to assess the procedure and product quality and identify weak links in the control and process, providing a framework for process optimization and improvements.

**Figure 4:**
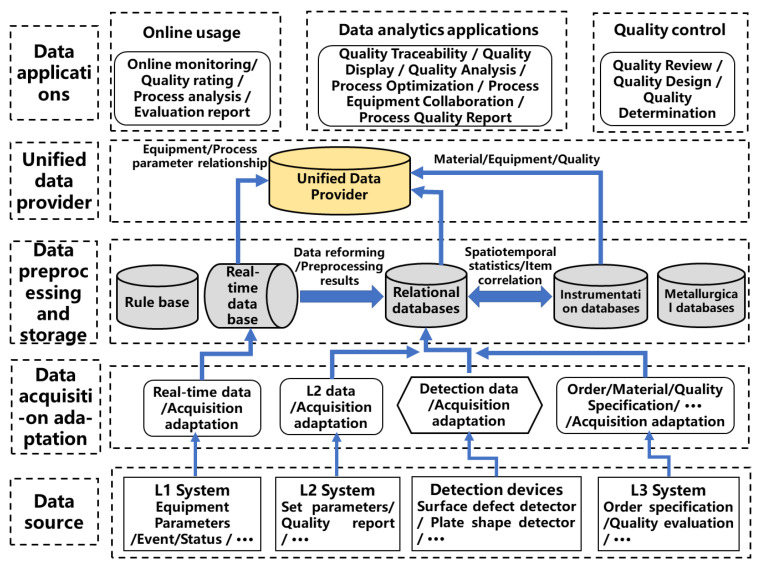
Architecture for quality control technologies in steel industry.

A schematic of the architecture for quality control technologies in the steel industry is shown in Figure 4. In the overall architectural design of the system, the complete process quality control system is divided into online and offline applications. On the one hand, the online application primarily provides collection, monitoring, early warning, and analysis functions with procedure characteristics, aimed at on-site quality inspectors and process technicians. This emphasizes the system’s real-time processing and timeliness, and provides quasi-real-time manufacturing process parameters, quality parameter determination, and early warning information to on-site operators and quality inspectors, thereby facilitating operations. On the other hand, offline application includes advanced analysis and application based on the product manufacturing process, emphasizing the entire process of quality data integration analysis from the manufacturing process’ point of view of product manufacturing procedure parameters, quality target parameters, quality inspection, and judgment results of traceability and analysis to solve cross-process, product manufacturing process system, and technical specifications.

### 4.3. Equipment Troubleshooting Technology

As a large-scale manufacturing enterprise, iron and steel enterprises have a large number of pieces of production equipment [102]. To begin with, the isolated equipment management information system results in data and functions overlapping between systems, each system’s inconsistent software and hardware design, and reduced data interchange and comprehensive analysis efficiency. As a result, the equipment management system and quality data in various operations have limited links. Data support for operations such as intelligent scheduling and quality analysis is also restricted. Moreover, the equipment maintenance plan is based on manual experience and normal maintenance, with no predictive maintenance. Additionally, the spare parts code does not correlate with a single product, making it impossible to implement the equipment’s whole life-cycle management. Consequently, equipment status control is highly reliant on the operators’ feeling of responsibility and expertise, restricting the equipment status online by monitoring objects and points. Furthermore, the interpretation of equipment status data is primarily based on manual experiences, with little knowledge accumulation and computer diagnosis abilities.

**Figure 5:**
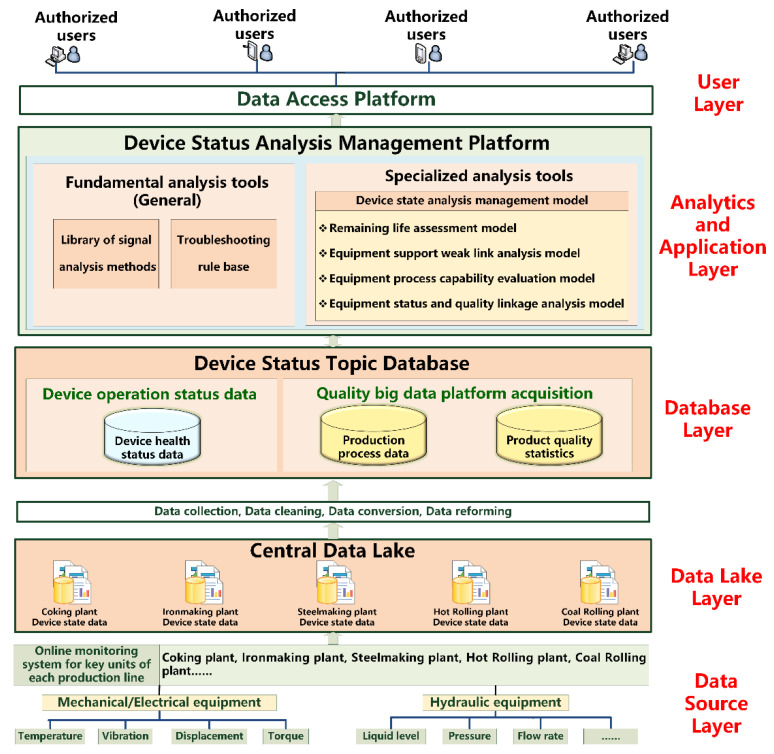
Architectural design for equipment troubleshooting systems in China’s steel industry.

In terms of equipment management, the next primary development direction is to provide a platform for equipment life-cycle management, remote status online monitoring, and intelligent diagnostics of critical equipment [103,104,105]. Figure 5 depicts an architectural design for equipment troubleshooting systems in China’s steel industry. The equipment theme database was created by combining big data analysis with data from the manufacturing process and product quality, as well as current spare parts procurement by maintenance management and functional correctness verification. As a result, predictive maintenance management, equipment functional accuracy and product quality linkage analysis, dynamic optimization of spare parts procurement strategy, equipment life-cycle management, and evaluation were used to achieve equipment status visualization and real-time early warning. This could effectively increase overall equipment efficiency and decrease average failure interval time, decrease average maintenance time, and offer equipment and technical support for upgrades.

### 4.4. Intelligent Machinery

Intelligent machinery with capabilities such as self-detection, self-diagnosis, and self-regulation should be widely used [106,107,108,109], especially in dangerous positions that involve high temperature, high coal gas, and repeated labor, to achieve accurate control in operations during ironmaking and steelmaking procedures; minimize staff labor intensity; and increase production efficiency and quality stability in the steel industry. The steel industry’s intelligent machinery is largely concerned with four aspects.

- Intelligent logistics equipment, such as an AGV (automated guided transport vehicle), unmanned elevators, intelligent roller rooms, intelligent three-dimensional factories, and flat storage facilities;
- Industrial robots including automatic slag fishing robots, automatic slag cleaning robots, intelligent temperature measurement robots, intelligent inspection robots, automatic baling robots, automatic coding robots, automatic alignment devices, automatic loading and unloading devices, and automatic welding devices;
- Intelligent detection equipment, which includes, in addition to the previously mentioned key component detection technology, intelligent monitoring of personnel safety, intelligent monitoring of safety facilities, and intelligent monitoring of equipment operational status; eddy current flaw detector; particle detector; thickness gauge; convexity meter; plate roller; and product contour detection device;
- Advanced control technology, one-key intelligent control technology of steelmaking, converter automatic steel production technology, refining process automatic control system, plate-type intelligent control technology, and other process intelligence and refined control technology.

## 5. Conclusions

This study introduced and summarized China’s steel industry’s intelligent manufacturing in recent years. To begin, this study defined intelligent manufacturing’s main aim as enhancing productivity, improving product quality, decreasing production costs, and shortening product development cycles. Then, the layers of the universal framework for intelligent manufacturing in China’s steel industry were divided into the device layer (DL), network layer (NL), big data layer (BDL), and application layer (AL). Furthermore, intelligent manufacturing in China’s steel industry was generally described, and two separate typical models of intelligent manufacturing including the rolling process and steelmaking and rolling process were offered. Finally, critical intelligent manufacturing technologies such as online detection technologies, quality-controlling technology for the entire procedures of steel industry, equipment troubleshooting technology, and intelligent machinery have been specifically introduced. This research not only helps to comprehend the development model, essential technologies, and construction techniques of intelligent manufacturing in China’s steel industry, but also provides vital inspiration for the manufacturing industry’s digital and intelligence updates and quality improvement.

## Figures and Tables

**Table 1:** Main intelligent manufacturing project in steel industry of China.

Project Name	Main Production Lines	Enterprise	Time/Year
Digital metallurgical mine	Mine	Angang Mining Company	2015
Intelligent workshop for hot rolling	Rolling	Baosteel	2015
Intelligent factory for iron and steel enterprise	Steelmaking and rolling	Hesteel	2016
Intelligent factory for silicon steel in the cold rolling process	Rolling	Shougang	2016
Intelligent manufacturing of high-precision special steel wire	Rolling	Shengtong Steel	2017
Digital workshop for cold rolling	Rolling	Baosteel	2017
Digital workshop for stainless steel in cold continuous rolling	Rolling	Taisteel	2017
Intelligent manufacturing in the whole process of high-end wire rod	Steelmaking and rolling	ShaSteel	2017
Intelligent factory for seamless steel pipe	Pipe Rolling	Hengyang Valin Steel Pipe	2018
Intelligent manufacturing for steel plate	Plate Rolling	Nangang	2018
Intelligent manufacturing for steel thick plate	Plate Rolling	Angang Steel	2018

**Table 2:** Main sensors used for intelligent manufacturing in steel industry.

Sensor Types	Sensor Names
**General sensors**	Material composition detector, flue gas composition detector, material particle size detector, temperature detector, flow meter, pressure gauge, gas alarm, spectrum analyzer, fluorescence analyzer, etc.
**High-temperature application sensors**	Furnace melt pool height detection, furnace hot field image recognition, solid material automatic sampling analysis, material surface height online detection, material pile morphology automatic monitoring, melt temperature online detection, furnace temperature online detection, melt composition online detection, flame morphology online detection, high-temperature flue gas online detection, etc.
**Rolling testing sensors**	Rolling pressure, rolling time, steel surface temperature, vibration signal, hydraulic signal, motor signal, etc.
**Quality testing sensors**	Surface defect detection, steel roughness, steel dimensions, steel thickness, internal defect detection, mechanical property testing, stress testing, welding performance testing, etc.
